# Enterococcal Endocarditis: Hiding in Plain Sight

**DOI:** 10.3389/fcimb.2021.722482

**Published:** 2021-08-30

**Authors:** Aaron M. T. Barnes, Kristi L. Frank, Gary M. Dunny

**Affiliations:** ^1^Department of Microbiology and Immunology, University of Minnesota School of Medicine, Minneapolis, MN, United States; ^2^Department of Laboratory Medicine and Pathology, University of Minnesota School of Medicine, Minneapolis, MN, United States; ^3^Department of Microbiology and Immunology, Uniformed Services University of the Health Sciences, Bethesda, MD, United States

**Keywords:** biofilm, infection source, microbial adherence, host colonization, immune evasion

## Abstract

*Enterococcus faecalis* is a major opportunistic bacterial pathogen of increasing clinical relevance. A substantial body of experimental evidence suggests that early biofilm formation plays a critical role in these infections, as well as in colonization and persistence in the GI tract as a commensal member of the microbiome in most terrestrial animals. Animal models of experimental endocarditis generally involve inducing mechanical valve damage by cardiac catheterization prior to infection, and it has long been presumed that endocarditis vegetation formation resulting from bacterial attachment to the endocardial endothelium requires some pre-existing tissue damage. Here we review both historical and contemporary animal model studies demonstrating the robust ability of *E. faecalis* to directly attach and form stable microcolony biofilms encased within a bacterially-derived extracellular matrix on the undamaged endovascular endothelial surface. We also discuss the morphological similarities when these biofilms form on other host tissues, including when *E. faecalis* colonizes the GI epithelium as a commensal member of the normal vertebrate microbiome - hiding in plain sight where it can serve as a source for systemic infection *via* translocation. We propose that these phenotypes may allow the organism to persist as an undetected infection in asymptomatic individuals and thus provide an infectious reservoir for later clinical endocarditis.

## Introduction

Enterococci are a paradox among bacteria: adaptable to a wide range of environmental conditions (pH, temperature, salinity, bile acids, etc.), resistant to numerous antibiotic compounds, and flexible enough to thrive as both common commensals and opportunistic pathogens across a range of clinical situations (Lebreton et al., 2014; Goh et al., 2017; Gaca and Lemos, 2019). As common commensals of the GI tract, they are part of our normal gut microbiota, but in the vasculature, enterococci are a leading cause of chronic (subacute) endocarditis - and both are particularly true for *Enterococcus faecalis* (Lebreton et al., 2014; Ch’ng et al., 2019; Ramos et al., 2020). Clinically, enterococci account for ~10% of valvular endocarditis cases; *E. faecalis* is the causative agent in the majority of these infections (Holland et al., 2016). The formation and development of bacterial biofilms – bacteria attached to a host surface and surrounded by a bacterially-derived extracellular matrix (ECM) – has been shown to occur during *E. faecalis* colonization of the murine GI tract (Barnes et al., 2017) and is also a significant pathogenic factor in animal models of both enterococcal catheter-associated urinary tract infection and endocarditis (Singh, 2007; Thurlow et al., 2010; Frank et al., 2013; Sillanpää 2013; Kafil, 2015; Madsen et al., 2017). This colonization leads to the development of a protective bacterial biofilm on the native or artificial tissue: the establishment of a biofilm frequently leads to markedly increased resistance to antimicrobial compounds (Holmberg and Rasmussen, 2016).

**Figure 1:**
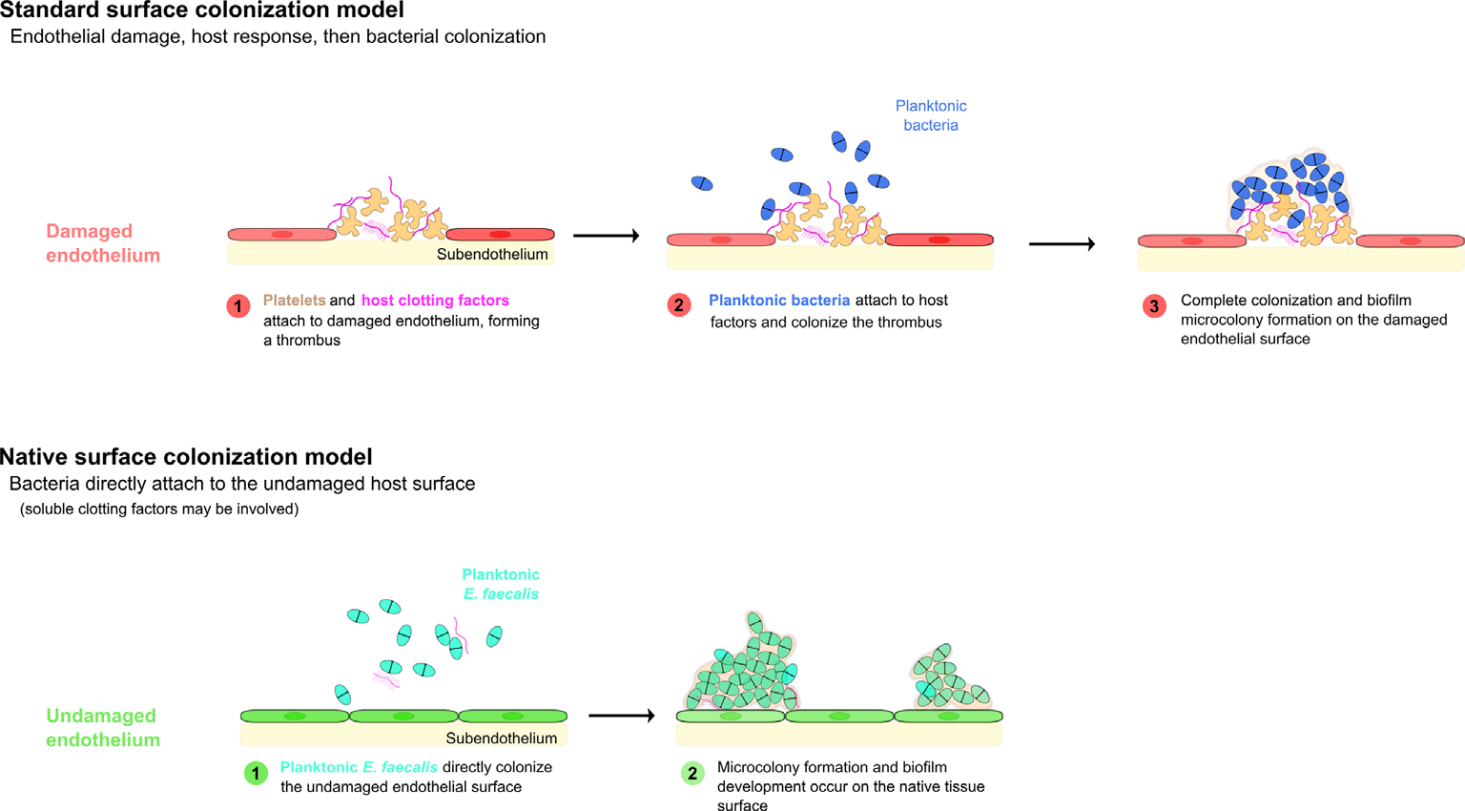
Models of bacterial endovascular colonization. Canonical models of endovascular colonization typically involve an initial inflammatory event and/or cell surface damage, which leads to host factor recruitment, platelet aggregation, and finally bacterial colonization of the nascent thrombus. Recent research suggests that enterococci (among other bacterial species) can attach to the undamaged endothelial cells in some model systems without the involvement of significant host factors (adapted from Barnes et al., 2021).

The classical or canonical model of bacterial endocarditis states that bacteria colonize a pre-existing, abacterial aggregation of host factors. Specifically, this model postulates a two-step process in which the accumulation of platelets, components of the coagulation cascade (fibrinogen, thrombin, etc.), and other host factors first occurs in response to an insult forming a “sterile vegetation”, then bacteria present in the bloodstream colonize this abnormal surface, forming a largely stationary nidus of infection (Fig 1; Holland et al., 2016).

We have previously shown that *E. faecalis* directly adheres to and colonizes the gut epithelial surface (Barnes et al., 2017), forming discrete biofilm microcolonies throughout the GI tract in a germ free mouse model. A similar pattern of native host surface colonization also occurs in a rabbit model of cardiac endovascular infection (Barnes et al., 2021). Combined with the lack of gross systemic host responses to this colonization over several weeks, and the ability of *E. faecalis* to attach to undamaged endothelium, these findings support a hypothesis in which attachment of enterococci to the cardiac endothelium plays a similar role in the prelude to pathogenic endocarditis as it does in non-pathogenic gut epithelial colonization.

Here we review both the recent and historical evidence for this type of colonization, demonstrate the ways in which the canonical model does and does not align with the recent discoveries in the field, and discuss the potential paths forward to better understand the pathophysiology surrounding this increasingly important clinical infection.

## Background

*Enterococcus faecalis* – originally described in 1906 as *Streptococcus faecalis* before being transferred to the genus *Enterococcus* in 1984 – has been recognized as a causative agent in endocarditis since its original English publication (Andrewes and Horder, 1906). (For a thorough review of the original clinical identification and publication of the bacteria eventually labeled as the genus *Enterococcus*, see Lebreton et al., 2014).

As noted above, the classical model of bacterial colonization of the heart involves an abiotic accumulation of host factors, typically associated with an endovascular insult. Notably, however, numerous papers in the older (pre-1975) literature reported that enterococcal endocarditis appears to occur in a significant fraction of patients without obvious pre-existing gross endothelial damage or cardiac structural defects (Geraci and Martin, 1954; Toh and Ball, 1960). (As often occurs in older literature, the precise identification of specific bacterial species can be difficult to determine definitively.) These clinical findings have also been reported in several animal model systems, including pigs (Jones, 1969) and rabbits (Durack et al., 1973).

Through the 1970s and 1980s, the focus of the medical community on enterococci was mainly due to the high degree of intrinsic and transmissible antibiotic resistance in these bacteria relative to routinely encountered pathogenic streptococci [note that enterococci were classified phylogenetically as members of the genus *Streptococcus* until the 1980s (Schleifer and Kilpper-Bälz, 1984)]. Genetic and molecular analysis of plasmids and transposable elements during this time period provided an important experimental foundation for future genome-wide analyses of enterococcal virulence (Clewell, 1990; Murray, 1990). However, the overall clinical incidence of confirmed enterococcal infections remained low during much of this period, though whether this reflected a true incidence rate or was simply a function of a more limited diagnostic environment is unclear.

During the 1980s, the increasing use of oral prophylaxis with cephalosporins contributed to the emergence of enterococci (mainly *E. faecalis*) as major hospital pathogens, with specific genotypes capable of epidemic spread, nationally and internationally (Donati et al., 1993; Pallares et al., 1993; Peng et al., 1994; Nicoletti and Stefani, 1995; de Vera and Simmons, 1996; Gin and Zhanel, 1996). Beginning in the 1990s, these clinical developments accelerated systematic efforts to identify critical genetic determinants of virulence in nosocomial- and other opportunistic enterococcal infections. Seminal studies in this effort focused on the identification of enterococcal antigens to which patients suffering from infections mounted an antibody response (Shorrock et al., 1990; Xu et al., 1997; Rich et al., 1999; Teng et al., 2002; Nallapareddy et al., 2006). Many of the relevant antigens identified in these early studies were surface-exposed components of the enterococcal cell envelope (Ebp, Ace, Epa). Follow up studies confirmed important functions of these components in adherence to host tissues and virulence and using *in vitro* assays, as well as animal infection models including experimental endocarditis. In addition to the aforementioned adherence factors, which are chromosomally encoded, evidence for contributions of plasmid-encoded surface adhesins, such as Aggregation Substance (Galli et al., 1989; Olmsted et al., 1991; Hirt et al., 1993; Dunny et al., 1995), to host colonization also accumulated during the same time frame (Chow et al., 1993; Olmsted et al., 1994; Schlievert et al., 1998).

Over the past twenty years, enterococci have continued their emergence as important healthcare-associated pathogens. This development is likely multifactorial and has been driven, in part, by improved diagnostics, an aging population, increasingly invasive medical procedures, and further spread of antibiotic resistance (Fernández-Hidalgo et al., 2020; Pericàs et al., 2020; Shah et al., 2020; Escolà-Vergé et al., 2021). The same time period also marked an explosion of research in the general field of bacterial biofilms (Parsek and Fuqua, 2004; Haussler and Parsek, 2010; Haussler and Fuqua, 2013; Visick et al., 2016; Høiby, 2017; Fuqua et al., 2019), the publication of full genome sequences for *E. faecalis* V583 (Paulsen et al., 2003), OG1RF (Bourgogne et al., 2008), and dozens of other strains (Palmer et al., 2010), as well as the development of improved tools for the genetic study of *E. faecalis* (Kristich et al., 2005; Kristich et al., 2007; Kristich et al., 2008; Ballering et al., 2009). As a result, our understanding of the genetic basis of biofilm formation in *E. faecalis* during both *in vitro* growth and infection significantly expanded (Mohamed and Huang, 2007; Paganelli et al., 2012; Dunny et al., 2014; Ch’ng et al., 2019; Tan et al., 2020). Our group’s transposon mutagenesis and recombinase-based *in vivo* expression technology (RIVET) genetic screens yielded non-overlapping but complementary results that identified multiple determinants of *in vitro* biofilm formation in the chromosome of strain OG1RF (Kristich et al., 2008; Ballering et al., 2009). The promoters of twenty-eight of the genes identified in these *in vitro* screens (2 from transposon screen, 26 from RIVET screen) were also found when the same RIVET library was screened in a rabbit model of subcutaneous implanted foreign body infection (Frank et al., 2012). However, when ten strains with mutations in biofilm-associated genes from these screens were tested for *in vivo* virulence defects in a rabbit model of infective endocarditis, only two genes (*ahrC* and *eep*) were found to have significant roles in endocarditis pathogenesis (Frank et al., 2012; Frank et al., 2013). The conclusion that *in vitro* biofilm phenotypes do not strongly correlate with infective endocarditis was further supported by the findings of Leuck et al., who showed that *E. faecalis* clinical isolates classified as poor biofilm formers in a standard *in vitro* microtiter dish assay colonized porcine heart valves in an *ex vivo* assay as well as strong biofilm-forming clinical isolates (Leuck et al., 2014).

Readers are referred to Madsen et al. for a summary of nine *E. faecalis* infective endocarditis virulence factors, as identified through a systematic literature review performed by those authors (Madsen et al., 2017). These virulence factors include aggregation substance, hemolysin, cell wall glycolipids, stress protein gls24, the Ebp pili proteins, secreted protease GelE, membrane metalloprotease Eep, and the adhesins Ace and EfbA (Madsen et al., 2017). A tenth *E. faecalis* endocarditis virulence factor is the transcriptional regulator AhrC (Frank et al., 2013), which influences expression of the *ace* and *ebp* genes (Manias and Dunny, 2018).

## Drivers of *E. faecalis* Bacteremia

Bacteremia is clearly a prerequisite for the development of bacterial colonization of the endothelium and infectious endocarditis. The original nidus of infection in acute bacterial endocarditis is often identifiable, a fact made easier by the relatively short time span between bacteremia and frank endocarditis. Chronic endocarditis - like typical enterococcal endocarditis - is often much less clear (Milbrandt, 1998). Many sources have been postulated, ranging from oral colonization in endodontic disease to translocation of commensal enterococci in the GI tract. Due partially to their adaptations to hard environmental conditions, enterococci are also the second most common cause of nosocomial bacteremia: exogenous infection *via* contaminated environmental surfaces in healthcare settings can lead to direct seeding of the vasculature (catheterization, contamination of implantable medical devices, etc.) or indirectly *via* colonization of the urinary or GI tracts (Arias and Murray, 2012). Endogenous infections also occur *via* translocation across the GI tract epithelium - a process that is exacerbated by common antibiotic treatments that can dramatically enrich the proportion of enterococci in the gut microbiome (Dubin and Pamer, 2014). Translocation of *E. faecalis* across the GI tract epithelial barrier and subsequent invasion into the circulatory system was experimentally demonstrated in a murine model over 30 years ago (Wells et al., 1990), with more recent work extending these findings and identifying invasion-defective *E. faecalis* mutant strains in a T84 cell culture model (Zeng et al., 2004) and high-resolution imaging of the process with additional insights into intracellular invasion (Archambaud et al., 2019). Notably, while enterococci are also common residents of the oral cavity and an important cause of endodontic disease – and despite the persistent expectation that oral enterococci would turn out to be a common nidus for endocarditis – cohort analysis has not shown oral cavity infections to be common factors in IE. For example, in a recent large cohort Spanish study comparing enterococcal IE (516 patients) and non-enterococcal IE cases (3,308 patients), only 1.6% of enterococcal cases could be assigned an oral origin *vs* 6.7% of non-enterococcal cases (Pericàs et al., 2020).

In addition to organism-specific translocation possibilities, it is also possible that sufficient GI barrier disruption occurs under severe physiologic stress to allow for bacterial invasion through systemic host immunosuppression (Manfredo Vieira et al., 2018; Fine et al., 2020; Little et al., 2020). Notably, it remains an open question whether enterococcal translocation occurs as a consequence of this host immunosuppression or if enterococci can drive the suppressive process and are thus immunomodulatory themselves (Fine et al., 2020). GI barrier disruption and bacterial translocation can also be driven pharmacologically with common antibiotics at clinically-relevant dosing in a mouse model - after only a single dose in some cases - with *E. faecalis* again playing a prominent role (Knoop et al., 2016).

While limited clinical data are available so far, there is also some recent evidence for a correlation between enterococcal endocarditis cases and cryptic colon cancers (Khan et al., 2018; Cabiltes et al., 2020; Pericàs et al., 2021) - whether this is a significant relationship between these disparate clinical entities (as in the majority of *Streptococcus gallolyticus* subsp. *gallolyticus* – formerly *Streptococcus bovis* biotype I (Pasquereau-Kotula et al., 2018) – endocarditis cases) remains unclear. Other disease states have also been shown to be related: Stanley et al. reported in a 2016 paper that a murine model of ischemic-reperfusion stroke revealed bacteremia by a select group of commensal bacterial strains, of which enterococci were the most abundant (Stanley et al., 2016).

## Enterococci and Colonization of the Epithelial Cell Surface

While the standard model of bacterial endocarditis development involves the initial formation of a host-derived thrombus followed by the colonization of this thrombus by bacteria present in the bloodstream, there are several examples of bacteria that have been reported to directly colonize host epithelial surfaces - and, in fact, this direct attachment mode of colonization may be more common than is generally appreciated. Among the bacterial species that have been shown to attach directly to the endothelium under at least some condition, *S. aureus* is perhaps the most well studied example. Pappelbaum et al. demonstrated that *S. aureus* attachment to intact endothelial cells is increased (and perhaps mediated by) ultra-large von Willebrand factor - a host cofactor we will consider further below (Pappelbaum et al., 2013).

Endothelium is specialized epithelium: while there has been some uncertainty around the specifics of endocardial development, it seems likely that the endocardium itself is modified endothelium (Dyer and Patterson, 2010; Harris and Black, 2010). *E. faecalis* is also able to colonize a variety of host epithelial surfaces directly in a number of different animal model systems. Barnes et al. demonstrated that *E. faecalis* can directly colonize the normal, undisturbed gut epithelial surface in a germ-free mouse model (Barnes et al., 2017). We also recently demonstrated enterococcal colonization of the endocardial and endovascular surfaces using a rabbit endocarditis model without a requirement for host tissue disruption or even limited surgical interventions (Barnes et al., 2021). Further support for a non-valvular colonization role in early endocardial colonization was reported by Thurlow et al., as cardiac tissue homogenates still showed grossly-elevated bacterial loads even after removal of the infected valve in their model (Thurlow et al., 2010). Finally, recent work by Brown et al. demonstrated in a mouse model system that peritoneal inoculation of *E. faecalis* can lead to sub-endothelial cardiac microlesions (Brown and Garsin, 2020; Brown et al., 2021). Of note, there was a brisk immune response to infection in this model, suggesting that the variations in the route of inoculation may lead to markedly different outcomes, both for the host and for the colonizing bacteria.

## Discussion

Clinically, the pathophysiology of infectious endocarditis (IE) is focused on the functional changes driven by bacterial damage to the cardiac valves, a process that is generally presumed to follow a predictable pathway from the deposition of host factors at an area of endocardial surface damage or insult, formation of a vegetation, valvular insufficiency, and a decrease in cardiac function. Staphylococci or streptococci are the most common causative agents in clinical cases of acute infective endocarditis, typically with rapidly-evolving, febrile presentations. Chronic (subacute) IE is more often associated with a slowly-evolving, insidious process with prodromal malaise and non-specific findings: oral streptococci and enterococci are the most common etiologies in these cases (McDonald, 2009). While overall rates of bacterial endocarditis in modern healthcare systems are stable or declining, the proportion of cases due to enterococci is increasing for complex reasons noted above (Dahl et al., 2019; Escolà-Vergé et al., 2021).

Since the 1970s, a significant fraction of both the basic and clinical research in the endocarditis literature has assumed that physical damage of the vascular endothelium is a requirement for the active development of IE, with most models based on an initial host response (platelets, soluble components of the coagulation cascade, etc.) followed by bacterial colonization of the nascent thrombus (Keynan and Rubinstein, 2013). Close review of the historical, pre-1975 literature, however, reveals that IE has been reported in numerous, diverse animal models without this damage (Wadsworth, 1919; Jones, 1969; Vakilzadeh et al., 1970; Jones, 1972). In particular, authors specifically note that, while pre-infection mechanical ablation of the endothelium increases the rates of vegetation formation (and thus decreases the number of animals needed for a given set of experiments), this is a function of efficiency and convenience, not a biological requirement (Durack and Beeson, 1972a; Durack and Beeson, 1972b; Durack et al., 1973).

**Figure 2:**
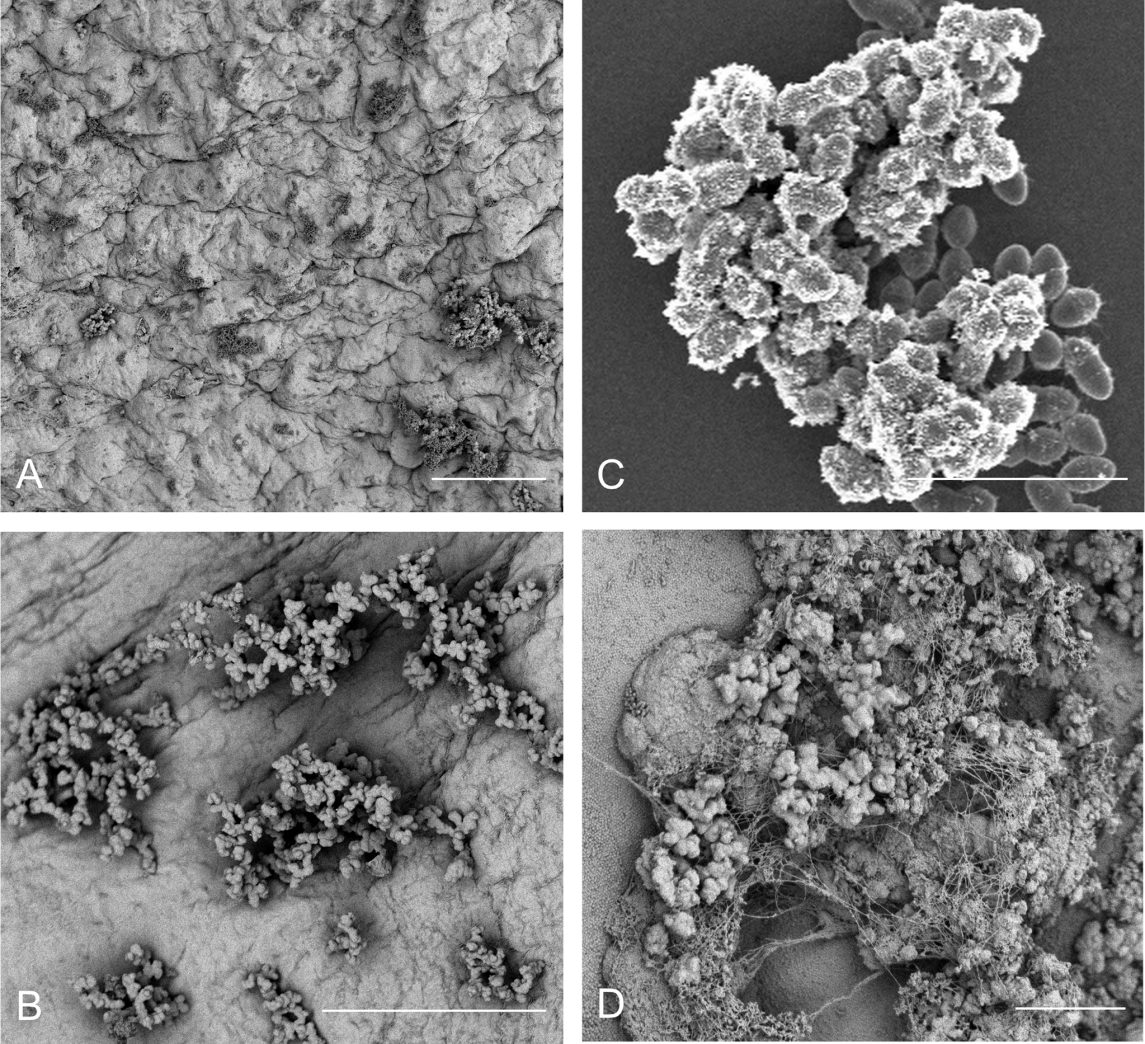
*E. faecalis* attachment, microcolony formation, and biofilm development on a range of *in vitro* and *in vivo* substrates demonstrates morphological conservation. **(A)** OG1RF attachment and microcolony development in a leporine model of endovascular infection (96 hr) with pre-inoculation mechanical damage to the aortic valve *via* catheterization (bar = 50 μm); **(B)** OG1RF attachment and microcolony development in a leporine model of endovascular infection (96 hr) from an uncatheterized rabbit (bar = 50 μm); **(C)** *E. faecalis* OG1RF microcolony formation *in vitro* on an Aclar fluoropolymer membrane (8 hr; bar = 5 μm). **(D)** OG1RF colonization of the GI tract in a germ-free murine model (72 hr; bar = 5 μm).

Thus, while it is reasonable to presume that structural cardiac abnormalities or pre-existing disturbances to the cardiac endothelium in human patients almost certainly contribute to increases in bacterial colonization rates and eventual endocarditis, there seems to be little actual evidence that frank damage to the endothelial surface is an actual requirement for bacterial colonization – a fact that has also been previously reported (Tunkel and Scheld, 1992). However, even in reported models in which no pre-inoculation endothelial damage has been incorporated, the process of bacterial colonization is still regarded as requiring an established, host-derived thrombus as a prerequisite (McDonald, 2009). As previously noted, there are several important pathogens that appear to be able to directly colonize the endothelial surface under some circumstances (Holland et al., 2016, **Figure 1**). We recently reported the direct colonization of the undamaged endothelial surface by *E. faecalis* in a rabbit model system of endocarditis without any apparent host factor involvement (Barnes et al., 2021) (**Figures 2A, B**). Notably, the endothelial colonization we reported there was focused on non-valvular microcolony formation and biofilm development as a bacterial strategy for persistent infections rather than the classic frank valvular endocarditis: while there is no reason to suspect enterococcal attachment on the cardiac valve surfaces would be strikingly different, further characterization of that etiology will be important. It is also important to note that the endothelial colonization and establishment of a biofilm population on the valvular surfaces may be temporally separate, further suggesting that a putative GI source of enterococcal bacteremia may proceed through several stages prior to the manifestation of clinical endocarditis symptoms.

But how do enterococci interact with the surface of normal cells rather than the exposed sub-endothelial components, platelets, and fibrin seen in classical endocarditis models? One possibility in the vasculature is that – like *Staphylococcus aureus* and *Streptococcus pneumoniae* (among others) – enterococci bind to circulating von Willebrand factor (vWF; Steinert et al., 2020). vWF is a critical component of hemostatic pathways in vertebrates (Wagner, 1990), and *E. faecalis* strain OG1RF does contain virulence factors (ElrA) that appear to interact with vWF domains (Jamet et al., 2017). In this model, circulating vWF binds to planktonic bacteria which, in turn, bind to surface-attached vWF on endothelial cells, allowing them to anchor to the cell surface. This “cloaking” in host vWF is also proposed as the mechanism behind interfering with platelet recruitment and other host coagulation cascade responses. Alternatively, a recent report by Gaytán et al. demonstrated in multiple bacterial species that a novel sialic acid-binding adhesin was critical for infective endocarditis, though the relationship to enterococcal endocarditis is unclear (Gaytán et al., 2021). While we cannot rule out these host-factor interactions in enterococcal IE, we are struck by the marked morphological consistency between *E. faecalis* microcolony formation in the vasculature and other non-circulatory system models (murine GI tract, *in vitro* polymer surfaces, etc.) which suggests another, perhaps more universal attachment mechanism (**Figure 2**).

The possibility that patients with enterococcal endocarditis may infect themselves *via* GI translocation would obviate a number of issues in identifying the nidus of infection in many clinical cases: antibiotic and/or systemic stress-mediated gut permeability to enterococci are common conditions in both outpatient and inpatient settings. Additionally, the lack of an obvious systemic, cell-mediated immune response seen in some - though not all - endovascular infection models, suggesting that *E. faecalis* may be able to evade the host immune system for lengthy periods of time, adds a further wrinkle to establishing concrete relationships between the onset of (perhaps transient) bacteremia and endovascular colonization. This area of research clearly requires further investigation to understand both the potential and actual routes of patient self-infection.

The complex process of enterococcal biofilm induction – from surface adherence and attachment through microcolony maturation and the establishment of a chronic disease state – has only recently come under sustained scrutiny. While much of the historical *in vitro* work over the past decades has provided a solid understanding of the mechanisms of surface attachment, the role of enterococcal virulence factors, and the processes behind plasmid exchange and antibiotic resistance, how they work *in vivo* to cause disease is a matter of considerable debate. Additionally, many laboratory-based *in vitro* systems to study biofilm formation prove to be discordant with *in vivo* studies, suggesting the need for further refinements. Finally, the general mechanisms of biofilm formation in clinical disease states has been understudied – and endocarditis is no exception. As basic research over the past decade has shown *in vitro*, the genetic and physiologic components of biofilm development are likely to significantly differ between bacterial species: there is unlikely to be a universal biofilm inhibitor. While there may be commonalties between some species, the outliers are also important to study – a role enterococci have played for years. Clinically, roughly half of enterococcal IE cases fail to identify a definitive nidus. This new model of extended enterococcal microcolony persistence on the cardiac endothelium could be consistent with a cryptic enterococcal infection mechanism.

## Author Contributions

All authors contributed to the article and approved the submitted version. AB generated the figures. All authors reviewed drafts of the manuscript and made revisions.

## Funding

This work was supported by the National Institutes of Health grants GM049530, GM118079, AI120601, AI058134, and AI105454 to GD. AB received additional support *via* NIH training grants HL007741 and AI055433 for portions of this work. KF was supported by NIH/NIAID award AI141961, American Heart Association award 17SDG33350092, and Uniformed Services University award R0733973.

## Author Disclaimer

The opinions or assertions contained herein are the private ones of the authors and are not to be construed as official or reflecting the views of the Department of Defense, the Uniformed Services University of the Health Sciences, the National Institute of Allergy and Infectious Diseases, the National Institute of General and Medical Sciences, the National Institutes of Health, or any other agency of the U.S. Government, or the American Heart Association. The funding agencies had no role in study design, data collection and analysis, decision to publish, or preparation of the manuscript.

## Conflict of Interest

The authors declare that the research was conducted in the absence of any commercial or financial relationships that could be construed as a potential conflict of interest.

## Publisher’s Note

All claims expressed in this article are solely those of the authors and do not necessarily represent those of their affiliated organizations, or those of the publisher, the editors and the reviewers. Any product that may be evaluated in this article, or claim that may be made by its manufacturer, is not guaranteed or endorsed by the publisher.
